# Allopatric speciation in ticks: genetic and reproductive divergence between geographic strains of *Rhipicephalus (Boophilus) microplus*

**DOI:** 10.1186/1471-2148-9-46

**Published:** 2009-02-25

**Authors:** Marcelo B Labruna, Victoria Naranjo, Atilio J Mangold, Carolina Thompson, Agustín Estrada-Peña, Alberto A Guglielmone, Frans Jongejan, José de la Fuente

**Affiliations:** 1 Departamento de Medicina Veterinária Preventiva e Saúde Animal, Faculdade de Medicina Veterinária e Zootecnia, Universidade de São Paulo, São Paulo, SP, 05508-270, Brazil; 2 Instituto de Investigación en Recursos Cinegéticos IREC (CSIC-UCLM-JCCM), Ronda de Toledo s/n, 13005 Ciudad Real, Spain; 3 Instituto Nacional de Tecnología Agropecuaria, Estación Experimental Agropecuaria Rafaela, CC 22, CP 2300 Rafaela, Santa Fe, Argentina; 4 Facultad de Veterinaria, Universidad de Zaragoza, Miguel Servet 177, 50013-Zaragoza, Spain; 5 Utrecht Centre for Tick-borne Diseases (UCTD), Department of Infectious Diseases and Immunology, Faculty of Veterinary Medicine, Utrecht University, Yalelaan 1, 3584CL, Utrecht, The Netherlands; 6 Department of Veterinary Tropical Diseases, Faculty of Veterinary Science, University of Pretoria, Private Bag X04, 0110, Onderstepoort, South Africa; 7 Department of Veterinary Pathobiology, Center for Veterinary Health Sciences, Oklahoma State University, Stillwater, OK 74078, USA

## Abstract

**Background:**

The cattle tick, *Rhipicephalus (Boophilus) microplus*, economically impact cattle industry in tropical and subtropical regions of the world. The morphological and genetic differences among *R. microplus *strains have been documented in the literature, suggesting that biogeographical and ecological separation may have resulted in boophilid ticks from America/Africa and those from Australia being different species. To test the hypothesis of the presence of different boophilid species, herein we performed a series of experiments to characterize the reproductive performance of crosses between *R. microplus *from Australia, Africa and America and the genetic diversity of strains from Australia, Asia, Africa and America.

**Results:**

The results showed that the crosses between Australian and Argentinean or Mozambican strains of boophilid ticks are infertile while crosses between Argentinean and Mozambican strains are fertile. These results showed that tick strains from Africa (Mozambique) and America (Argentina) are the same species, while ticks from Australia may actually represent a separate species. The genetic analysis of mitochondrial 12S and 16S rDNA and microsatellite loci were not conclusive when taken separately, but provided evidence that Australian tick strains were genetically different from Asian, African and American strains.

**Conclusion:**

The results reported herein support the hypothesis that at least two different species share the name *R. microplus*. These species could be redefined as *R. microplus *(Canestrini, 1887) (for American and African strains) and probably the old *R. australis *Fuller, 1899 (for Australian strains), which needs to be redescribed. However, experiments with a larger number of tick strains from different geographic locations are needed to corroborate these results.

## Background

The cattle tick, *Rhipicephalus (Boophilus) microplus*, is distributed in tropical and subtropical regions of the world [1]. Infestations with *R. microplus *economically impact cattle industry by reducing weight gain and milk production, and by transmitting pathogens that cause babesiosis (*Babesia bovis *and *B. bigemina*) and anaplasmosis (*Anaplasma marginale*) [2,3]. The morphological and genetic differences among *R. microplus *strains have been documented in the literature [4,5]. Although Londt and Arthur [6] argued that morphological differences between these strains are too slight to warrant species status, other results provided opposing evidence [7]. These authors demonstrated that hybrids resulting from crossing *R. (B.) microplus *strains from South Africa and Australia were sterile. Thus, *R. microplus *from South Africa and Australia might be regarded as different species [8].

Contradictory results have been reported after the analysis of different gene loci in *R. microplus *strains collected at different locations. It has been [9] found that the analysis of ribosomal internal transcribed spacer 2 (ITS2) sequences resulted in similar divergence between Australian and South African *R. microplus *strains and among tick strains from Australia, Brazil, Kenya and South Africa. However, a clear divergence in the acetylcholinesterase sequence between Australian and African/Mexican strains of *R. microplus *has been detected [10]. The mitochondrial 12S rDNA divergence between American and African *R. microplus *strains is lower than the divergence between these strains and the *R. microplus *from Australia and Nepal [11].

These results suggested that biogeographical and ecological separation between boophilid lineages may have resulted in different species of ticks in America/Africa and those from Australia, collectively known as *R. microplus*, but being actually separate species [11]. To test the hypothesis of the presence of different species in *R. microplus *ticks, herein we performed a series of experiments to characterize the the reproductive performance of crosses between ticks from Australia, Africa and America and the genetic diversity of *R. microplus *strains from Australia, Asia, Africa and America.

## Methods

### Tick crosses and fertility analysis

Tick crosses were conducted using strains originally determined as *R. microplus *from Argentina (ARG), Mozambique (MOZ) and Australia (AUS, Yeerongpilly strain) (Table 1). In experiment I, calves were infested with larvae of ARG and AUS, each strain separately infesting a cotton sleeve (20 cm diameter) glued (Kamar heat detector adhesive, Kamar, Steamboat Springs, CO, USA) to the shaved dorsum skin of two tick-naïve Holstein calves. After 12–15 days of infestation, sleeves were opened and engorged nymphs attached to the skin were manually removed from each sleeve and held in an incubator at 34°C and 95% RH for molting. Emerged adults, less than 24 hours from their ecdysis were then sorted to form the infestation male × female crosses into new sleeves glued to the dorsum of the same calves from where engorged nymphs were collected. Crosses were made with adult ticks from the same strain (AUS × AUS, and ARG × ARG; homologous crosses), and with adult ticks from different strains (AUS × ARG, and ARG × AUS; heterologous crosses) (Table 2). Each cross consisted of 15–20 males and 20–30 females per sleeve. Two additional sleeves, each containing only 20–30 unfed females from each strain were prepared to be the control of virgin females for each strain.

**Table 1:** Tick strains used in these studies.

**Tick species**	**Geographical origin**	**Collection date**	**Genbank accession No.**
*R. microplus*	Quimilí, Argentina	Apr-1998.	16S: EU918176
			12S: EU921758

*R. microplus*	Corichi Grande, Bolivia	Sep-1999	16S: EU918177
			12S: EU921759

*R. microplus*	Sao Gabriel, Brazil	Oct-2000	16S: EU918178
			12S: EU921760

*R. microplus*	Ciudad Quesada, Costa Rica	Sep-1998	16S: EU918179
			12S: EU921761

*R. microplus*	Asunción, Paraguay	Oct-1999	16S: EU918180
			12S: EU921762

*R. microplus*	Piura, Peru	Jun-2002	16S: EU918181
			12S: ND

*R. microplus*	Rocha, Uruguay	Jun-2002	16S: EU918184
			12S: EU921763

*R. microplus*	Australia (A). DDT-susceptible strain	Oct-1998	16S: EU918185
			12S: EU921767

*R. microplus*	Australia (B). DDT-resistant strain	Oct-1998	16S: EU918186
			12S: EU921768

*R. microplus*	Australia (C). Yeerongpilly strain	1950s	16S: EU918192
			12S: EU921769

*R. microplus*	Bourail, New Caledonia	2006	16S: EU918191
			12S: EU921770

*R. microplus*	Jakarta (Batavia), Indonesia	1951	16S: EU918189, EU918190
			12S: EU921771

*R. microplus*	Mozambique	2003	16S: EU918191
			12S: EU921766

*R. microplus*	Limpopo, South Africa	Jul-2003	16S: EU918182
			12S: EU921764

*R. microplus*	Tanzania	1973	16S: EU918183
			12S: EU921765

*R. microplus*	Izatnagar, India	2008	16S: EU918188
			12S: EU921770

*R. annulatus*	Egypt	1961	16S: ND
			12S: EU921773

*R. decoloratus*	Limpopo, South Africa	Jul-2003	16S: EU918193
			12S: EU921774

Abbreviation: ND, not done.

**Table 2:** Results of experiment I in which calves were infested with crosses of adult ticks of *Rhipicephalus (Boophilus) microplus *strains from Australia (AUS) and Argentina (ARG).

Cross^+^ (male × female)	Engorged female weight (mg)*	Feeding period (days)*	No. engorged females that oviposited (%)	Egg mass weight (mg)*	EPE*	% egg hatching*	F_1 _fertility^@^
ARG × ARG	267.5 ± 38.1 a	21.5 ± 0.5^&^a	26 (100)	129.4 ± 28.4 a, e	48.6 ± 8.9 a	93.1 ± 20.5 a	Yes
AUS × AUS	196.8 ± 43.1 b	19.8 ± 0.4^&^b	52 (83.9)	69.7 ± 25.6 b	34.3 ± 11.2 b	82.5 ± 25.2 a	Yes
AUS × ARG	264.0 ± 57.2 a	8.7 ± 1.6^#^c	9 (90.0)	121.6 ± 55.5 c, e	43.8 ± 15.1 a, b	36.0 ± 39.6 b	No
ARG × AUS	172.9 ± 34.8 c	9.0 ± 1.5^#^c	24 (100)	83.3 ± 23.5 c, f	47.9 ± 8.2 a	0.3 ± 0.5 c	**
___ × ARG	50.3 ± 55.8 d	15.3 ± 3.8^#^b, d	2 (50)	24.5 ± 26.2 a, b, d, f	24.0 ± 12.2 a, b, c	0.5 ± 0.7 c	**
___ × AUS	61.3 ± 40.0 d	16. ± 2.9^#^d	15 (65.3)	11.8 ± 7.4 d	13.3 ± 6.5 c	0.1 ± 0.3 c	**

^+^Each cross represented one independent infestation sleeve glued to the host dorsum.

* Values presented as mean ± standard deviation.

^&^Feeding period refers to days elapsed from larval infestation to detachment of engorged females.

^# ^Feeding period refers to days elapsed from adult infestation to detachment of engorged females.

EPE: egg production efficiency (weight of egg mass/weight of the engorged female × 100).

^@ ^Larvae generated from each cross were infested in a separate sleeve on a calf; fertility of the resulting engorged females was evaluated (see Table 4).

** Cross produced zero or a very low number of F_1 _larvae to perform an infestation.

Values followed by different letters in the same column are significantly different (*P *< 0.05).

All engorged females recovered from each sleeve (each separate cross) were individually weighed and left in an incubator at 25°C and 85% RH. The total egg mass produced by each female was weighed and its hatchability was determined as described [12]. The egg production efficiency (EPE) was determined as (weight of eggs/weight of the engorged female) × 100 [13].

In experiment II, tick-naïve New Zealand rabbits were infested with larvae from ARG, AUS, and MOZ strains inside a cotton sleeve (20 cm diameter) glued to the shaved dorsum skin of the rabbit. At 12–15 days post-infestation, sleeves were opened and engorged nymphs attached to the skin were manually removed from each sleeve and held in an incubator at 34°C and 95% RH for molting. Emerged adults, less than 24 hours from their ecdysis, were sexed to form the infestation male × female crosses in sleeves glued to the dorsum of a calf. A total of 12 sleeves were mounted on the calf to encompass the nine possible crosses between the three strains, as well as three additional sleeves for virgin control females for each strain (Table 3). Each cross consisted of 20–25 males and 25–30 females per sleeve, and procedures for recovery and analysis of engorged females recovered from each sleeve were as described for experiment I.

**Table 3:** Results of experiment II in which a calf was infested with crosses of adult ticks of *Rhipicephalus (Boophilus) microplus *strains from Australia (AUS), Argentina (ARG) and Mozambique (MOZ).

Cross^+^ (male × female)	Engorged female weight (mg)*	Feeding period (days)* ^#^	No. engorged females that oviposited (%)	Egg mass weight (mg)*	EPE*	% egg hatching*	F_1_fertility^@^
MOZ × MOZ	252.3 ± 81.1 a	7.6 ± 0.6 a, f	21 (96.1)	123.2. ± 60.0 a, c	47.1 ± 15.5 a, c, d, e	85.5 ± 30.4 a	Yes
ARG × ARG	257.4 ± 65.6 a	6.5 ± 0.8 b	19 (95.0)	131.9 ± 29.6 a	51.2 ± 5.7 a, c	85.3 ± 28.3 a	Yes
AUS × AUS	116.1 ± 36.3 b	7.4 ± 1.5 a, b, d	14 (100)	39.9 ± 17.9 b	33.4 ± 7.4 b	20.9 ± 28.0 b	Yes
MOZ × ARG	230.5 ± 58.2 a	8.1 ± 0.7 c, d, f	11 (91.7)	122.5 ± 36.2 a, c	50.2 ± 7.5 c, d	85.0 ± 31.7 a	Yes
MOZ × AUS	100.4 ± 32.4 b	8.8 ± 1.6 c	15 (83.3)	43.3 ± 18.3 b	39.9 ± 6.9 e	0.0 ± 0.0	**
ARG × MOZ	236.9 ± 58.9 a	9.2 ± 1.8 c, e	18 (100)	122.6 ± 38.2 a, c	51.0 ± 5.4 c	84.7 ± 33.3 a	Yes
ARG × AUS	117.8 ± 31.4 b	8.4 ± 1.1 c, d	10 (90.9)	55.6 ± 19.1 b	45.7 ± 6.6 d	0.0 ± 0.0	**
AUS × MOZ	226.2. ± 51.3 a	8.0 ± 1.3 c, f	24 (100)	103.9 ± 12.4 c	46.5 ± 12.4 c, d	53.6 ± 40.4 c	No
AUS × ARG	233.0 ± 56.6 a	7.0 ± 1.6 a, b	15 (83.3)	114.4 ± 30.0 a.c	47.5 ± 30.0 c, d	15.6 ± 21.5 b	No
___ × MOZ	96.0 ± 71.0 b, c	15.9 ± 3.2 g	11 (57.9)	61.4 ± 32.7 b	40.8 ± 13.9 b, d, e	0.0 ± 0.0	**
___ × ARG	66.2 ± 44.0 c, d	14.5 ± 3.3 g, h	8 (61.5)	21.1 ± 17.5 d	22.4 ± 9.9 f	0.0 ± 0.0	**
___ × AUS	52.9 ± 19.1 d	12.6 ± 1.2 h	7 (70)	8.3 ± 3.4 d	13.0 ± 4.7 g	0.0 ± 0.0	**

^+^Each cross represented one independent infestation sleeve glued to the host dorsum.

* Values presented as mean ± standard deviation.

^# ^Feeding period refers to days elapsed from adult infestation to detachment of engorged females.

EPE: egg production efficiency (weight of egg mass/weight of the engorged female × 100).

^@ ^Larvae generated from each cross were infested in a separate sleeve on a calf; fertility of the resulting engorged females was evaluated (see Table 4).

** Cross produced no F_1 _larvae to perform an infestation.

Values followed by different letters in the same column are significantly different (*P *< 0.05).

In order to verify successful reproductive compatibility between strains, unfed larvae (20–30 days old) obtained from the crosses (designated as F_1 _larvae) were used to infest a calf for experiment I-F_1 _ticks, and another calf for experiment II-F_1 _ticks (one sleeve used for infestation with larvae generated from each separate cross). The resulting engorged females were processed as previously described.

Feeding periods of ticks from each cross were compared by the non-parametric Mann-Whitney test, whereas engorged female weights and reproductive parameters (which show normal distribution) were compared by the Student *t-*test. Variables were considered significantly different if *P *< 0.05.

### Phylogenetic analysis of tick mitochondrial 12S and 16S rDNA

Specimens of sixteen strains of *R. microplus *from America, Africa, Asia and Oceania and one strain of each *R. annulatus *and *R. decoloratus *from Africa were used for DNA extraction and sequencing of mitochondrial 12S and 16S rDNA (Table 1). One to three specimens from each strain were analyzed. DNA was extracted from alcohol-preserved specimens and polymerase chain reaction (PCR) amplification of a fragment of the mitochondrial 16S rDNA was conducted as described [14]. The PCR conditions for 12S rDNA amplification were as described [15]. Amplified PCR products were purified using Wizard PCR Preps DNA Purification System (Promega Corporation, Madison, Wisconsin, USA). The purified DNA was directly sequenced at the IMyZA (Instituto de Microbiología y Zoología Agrícola, INTA, Castelar, Buenos Aires, Argentina). Both DNA strands were sequenced and assembled using BioEdit 7.05.3 software [16].

For phylogenetic analysis, the following tick mitochondrial 16S and 12S rDNA sequences available in the Genbank were also included:

#### 16S rDNA

*R. annulatus*, Spain (Z97877), *R. annulatus*, USA (L34311), *R. microplus*, USA (L34310), *R. microplus*, strain 9 from Taiwan (AY974232), *Dermacentor andersoni*, USA (L34299), *D. marginatus*, Spain (Z97879), *D. variabilis*, USA (L34300), *Haemaphysalis juxtakochi*, Uruguay (AY762323), *H. leporispalustris*, USA (L34309), *H. punctata*, Spain (Z97880), *Hyalomma dromedarii*, UK (L34306), *H. lusitanicum*, Spain (Z97881), *Rhipicephalus appendiculatus*, UK (L34301), *R. bursa*, Spain (Z97878), *R. pusillus*, Spain (Z97883) and *R. sanguineus*, Spain (Z97884).

#### 12S rDNA

*R. annulatus *(U95866), *R. annulatus*, Italy (AM410573), *R. annulatus*, Israel (AF133058), *R. microplus*, Nepal (AF150042), *R. microplus*, Australia (AF031847), *R. decoloratus*, Zimbabwe (AF150044), *R. decoloratus*, Kenya (AF031846), *R. kohlsi*, Jordan (AF150043), *R. kohlsi*, origin unknown (AY008686), *Dermacentor albipictus*, USA (AF150041), *D. andersoni*, USA (AF150040), *D. reticulatus*, France (AF150038), *Haemaphysalis leachi*, Zimbabwe (AF150035), *H. punctata*, Switzerland (AF150032), *Hyalomma dromedarii*, Morocco (AF150036), *H. truncatum*, Zimbabwe (AF150031), *Rhipicephalus appendiculatus*, Uganda (AF150028), *R. bursa*, Spain (AF150053), *R. evertsi evertsi*, Zimbabwe (AF150052), *R. pusillus*, France (AF150022), *R. sanguineus*, France (AF150020), and *R. turanicus*, France (AF150018).

Multiple sequence alignments were done using Clustal W [17]. Phylogenetic and molecular evolutionary analyses were conducted using MEGA version 4 [18]. Phylogenetic relationships between sequences were assessed by neighbored-joining (NJ) method [19]. The NJ topologies were examined using Tamura-Nei distances [20] and relative support for the internal nodes was tested by bootstrapping over 1,000 replications [21]. Tamura-Nei distance measures were used because this model corrects for multiple hits, taking into account the differences in substitution rate between nucleotides and the inequality of nucleotide frequencies. It distinguishes between transitional substitution rates between purines and transversional substitution rates between pyrymidines. All positions containing alignment gaps were eliminated only in pairwise sequence comparisons. Ticks species of the genus *Haemaphysalis *were treated as out-group.

### Analysis of microsatellite polymorphism

Microsatellite polymorphism was analyzed in tick strains from Argentina, Australia, Mozambique, India and New Caledonia (Table 1). For Argentina, Australia and Mozambique strains, female_strain 1 _× male_strain 2 _and female_strain 2 _× male_strain 1 _homologous and heterologous crosses were used. For each cross, egg masses from two separate crosses were used for genotyping. Tick DNA was obtained from egg batches (ARG, AUS and MOZ strains), pooled larvae (New Caledonia strain) or pooled whole ticks (India and Indonesia strains). Tick tissues were homogenized in liquid N or with a 1 ml tuberculin syringe with a 25-G needle to extract DNA with Tri Reagent (Sigma, St. Louis, MO, USA) following manufacturer's recommendations. The purified DNA was dissolved in distilled water and the concentration determined using the NanoDrop 1000 (Thermo Fisher Scientific, Wilmington, DE, USA).

Polymorphism was analyzed at microsatellite loci BmA12 (Genbank accession number DQ001904), BmA06 (DQ001905), BmB12 (DQ001906), BmC03 (DQ001907), BmC07 (DQ001909) and BmD12 (DQ001911) using oligonucleotide primers and PCR cycling conditions described [22]. The PCR was done with labeled forward primers (BmA12 and BmD12, 6-FAM; BmA06 and BmC07, NED; BmB12, VIC; BmC03, PET) in a 50-μl volume (1.5 mM MgSO_4_, 1X avian myeloblastosis virus (AMV) RT/*Thermus flavus *(*Tfl*) reaction buffer, 0.2 mM each deoxynucleoside triphosphate (dNTP), 5 u *Tfl *DNA polymerase, 0.2 μM of each oligonucleotide primer) employing the Access RT-PCR system (Promega, Madison, WI, USA). Reactions were performed in an automated DNA thermal cycler (Techne model TC-512, Cambridge, England, UK). The reaction was terminated after a final extension at 68°C for 5 min. Control reactions were done using the same procedures, but without DNA added to check contamination of the PCR reaction. PCR products were electrophoresed on 1% agarose gels to check the size of amplified fragments by comparison to a DNA molecular weight marker (1 Kb Plus DNA Ladder, Promega). Fragments were separated using an ABI 3730 automated DNA sequencer (Applied Biosystems, Inc. Foster City, CA, USA) and sized relative to a ROX-labeled internal size standard (GeneScan-500LIZ, Applera, Norwalk, CT, USA). The data were analyzed using program Peak Scanner (Applied Biosystems).

For statistical analysis, a binary matrix was constructed by scoring the different alleles from each microsatellite locus as presence (1) and absence (0) of the PCR bands for each cross. Based on the binary matrix, similarity matrixes were calculated using Jaccard's and Dice's coefficients [23,24]. The similarity matrix was subjected to Sequential Agglomerative Hieratical Nested Clustering (SAHN) and dendograms were constructed employing the Unweighted Pair Group Method of Arithmetic Averages (UPGMA) of Sneath and Sokal [25] to group the progenies into clusters. All the analyses were performed using statistical software package NTSYSpc version 2.01e [26].

## Results

### The crosses between Australia and Argentina or Mozambique strains are infertile

Tick crosses were conducted between AUS, MOZ and ARG *R. microplus *strains (Tables 2 and 3). Both homologous and heterologous crosses of ARG and MOZ produced fertile offspring. AUS ticks produced a viable, fertile offspring in homologous crosses. However, AUS females failed to yield viable larvae in heterologous crosses. Even though heterologous crosses of AUS males with ARG or MOZ females produced viable offspring (mean egg hatching varying from 15.6% to 53.6%), the resultant F_1 _hybrid ticks were infertile in all cases (Table 4). In contrast, F_1 _ticks from heterologous crosses between ARG and MOZ were as fertile as the F_1 _ticks from homologous crosses. This result suggested a lack of genetic compatibility between Australian ticks and strains from Africa and America. It was interesting to note the lower weight and feeding period of engorged AUS females, even in homologous crosses (Tables 2 and 4). Regardless of being fertile or not, females from all crosses were significantly heavier and showed feeding periods significantly shorter than their corresponding virgin female controls.

**Table 4:** Results of infestation with larvae obtained from each fertile cross in experiments I (Table 1) and II (Table 2) with *Rhipicephalus (Boophilus) microplus *strains from Australia (AUS), Argentina (ARG) and Mozambique (MOZ).

Experiment No. – Cross (male × female) that generated the larvae used for infestation	Engorged female weight (mg)*	Feeding period (days)* ^#^	No. engorged females that oviposited (%)	Egg mass weight (mg)*	EPE*	% egg hatching*
I – AUS × AUS	169.4 ± 37.3 a	20.2 ± 0.4 a	21 (70.0)	50.3 ± 20.9 a	29.5 ± 11.2 a	96.4 ± 11.0 a
I – AUS × AUS	192.8 ± 53.2 a, f	20.2 ± 0.4 a, b	5 (100)	52.0 ± 33.9 a, b	25.5 ± 10.8 a, d	27.2 ± 39.6 b
II – AUS × AUS	179.0 ± 19.7 b, g	21.1 ± 0.8 d, e	7 (87.5)	78.6 ± 18.7 b	43.5 ± 8.9 b	27.3 ± 37.2 b
I – ARG × ARG	278.7 ± 37.2 c	22.5 ± 0.9 f	30 (100)	140.7 ± 24.3 c	50.4 ± 5.2 b,c	95.8 ± 11.1 a,d
II – ARG × ARG	309.4 ± 26.0 d	24.6 ± 1.1 g	29 (96.7)	156.8 ± 20.0 d	50.8 ± 5.2 b,c	84.2 ± 28.3 c,d
II – MOZ × MOZ	374.1 ± 35.9 e	24.1 ± 1.3 c	28 (93.3)	183.3 ± 34.6 e	49.2 ± 9.4 b,c	78.8 ± 24.3 c
I – AUS × ARG	214.0 ± 55.4 f,g	20.6 ± 0.6 b.d	29 (96.7)	74.2 ± 25.3 b	33.2 ± 17.7 a	0.0 ± 0.0
I – AUS × ARG	257.6 ± 37.6 b	21.1 ± 1.3 e	30 (100)	62.0 ± 28.4 a,b	24.4 ± 11.3 a,d	0.0 ± 0.0
II – AUS × ARG	311.7 ± 29.1 b	21.0 ± 1.1 e	30 (100)	163.7 ± 24.5 d,f	52.4 ± 4.7 c,e	0.0 ± 0.0
II – AUS × MOZ	304.0 ± 36.4 a	20.1 ± 1.0 a	30 (100)	66.3 ± 35.0 a,b	22.3 ± 12.1 d	0.0 ± 0.0
II – MOZ × ARG	265.1 ± 58.8 e	24.0 ± 1.2 c	30 (100)	147.3 ± 38.6 c,d	55.6 ± 10.3 e,f	95.0 ± 17.6 a,d
II – ARG × MOZ	299.7 ± 57.8 h	23.6 ± 1.3 h	29 (97.7)	179.6 ± 44.1 e,f	58.7 ± 6.7 f	92.2 ± 14.7 a,c

* Values presented as mean ± standard deviation.

^# ^Feeding period refers to days elapsed from larval infestation to detachment of engorged females.

EPE: egg production efficiency (weight of egg mass/weight of the engorged female × 100).

Values followed by different letters in the same column are significantly different (*P *< 0.05).

### The Australian tick strain is genetically more divergent than Asian, African and American strains

**Figure 1:**
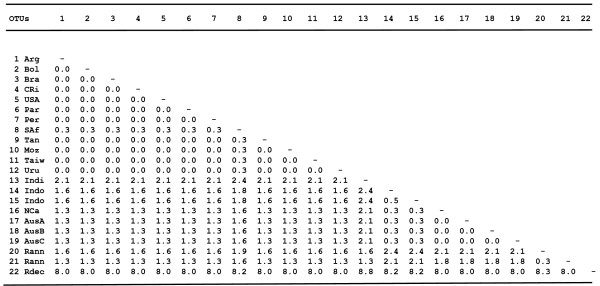
**Matrix of sequence differences on pairwise comparisons of the mitochondrial 16S rDNA gene for 19 *R. microplus *strains, *R. annulatus *and *R. decoloratus***. Proportion of nucleotide differences (10^2^) (*p*-distance) are shown in the lower left matrix. It is obtained by dividing the number of nucleotide differences by the total number of nucleotides compared. (OTUs Operational taxonomic units, 1 Argentina, 2 Bolivia, 3 Brazil, 4 Costa Rica, 5 USA L34310, 6 Paraguay, 7 Peru, 8 South Africa, 9 Tanzania, 10 Mozambique, 11 Taiwan AY974232, 12 Uruguay, 13 India, 14 Indonesia 1, 15 Indonesia 2, 16 New Caledonia, 17 Australia A, 18 Australia B, 19 Australia C, 20 *R. annulatus *Z97877, 21 *R. annulatus *L34311, 22 *R. decoloratus*).

**Figure 2:**
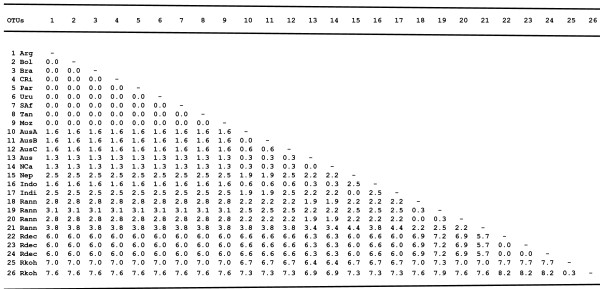
**Matrix of sequence differences on pairwise comparisons of the mitochondrial 12S rDNA gene for 17 *R. microplus *strains, *R. annulatus*, *R. decoloratus *and *R. kohlsi***. Proportion of nucleotide differences (10^2^) (*p*-distance) are shown in the lower left matrix. It is obtained by dividing the number of nucleotide differences by the total number of nucleotides compared. (OTUs Operational taxonomic units, 1 Argentina, 2 Bolivia, 3 Brazil, 4 Costa Rica, 5 Paraguay, 6 Uruguay, 7 South Africa, 8 Tanzania, 9 Mozambique, 10 Australia A, 11 Australia B, 12 Australia C, 13 Australia AF031847, 14 New Caledonia, 15 Nepal AF150042, 16 Indonesia, 17 India, 18 *R. annulatus *Egypt, 19 *R. annulatus *AM410573, 20 *R. annulatus *AF133058, 21 *R. annulatus *U95866, 22 *R. decoloratus *South Africa, 23 *R. decoloratus *AF150044, 24 *R. decoloratus *AF031846, 25 *R. kohlsi *AY008686, 26 *R. kohlsi *AF150043).

Approximately 400 bp of the mitochondrial 16S rDNA gene were successfully sequenced from specimens of *R. microplus *representing 16 different strains from America, Africa, Asia and Oceania. The analysis of 16S rDNA sequences variation revealed very little genetic diversity (0.0 to 0.3%) between American and African strains (Fig. 1). In contrast, genetic diversity at this locus was 1.3–1.6% and 1.6–2.4% when these strains were compared to *R. microplus *from Oceania and Asia, respectively (Fig. 1). For 12S rDNA, approximately 350 bp were sequenced from *R. microplus *representing 15 strains from America, Africa, Asia and Oceania. No differences were observed in 12S rDNA sequences between American and African strains (Fig. 2). However, when these strains were compared to *R. microplus *from Oceania and Asia, the genetic diversity was 1.3–1.6%, and 1.6–2.5%, respectively.

**Figure 3:**
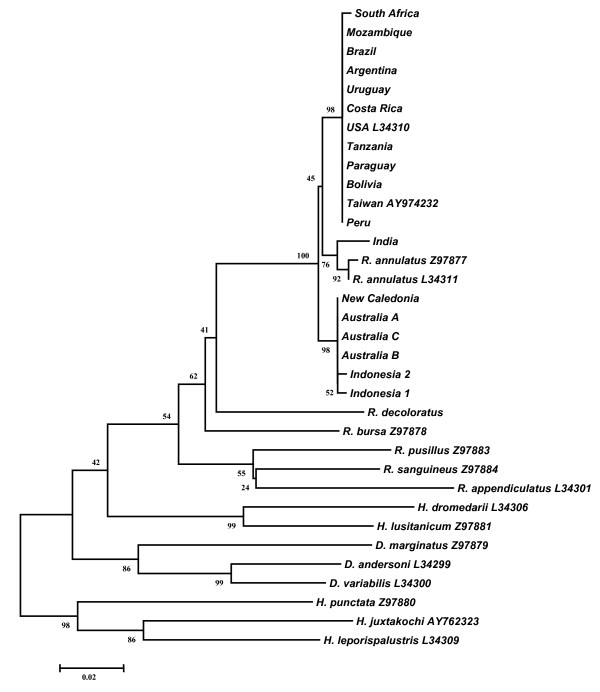
**Neighbor-Joining tree of the 16S rDNA sequences using the Tamura-Nei method**. The percentage of replicate trees in which the associated taxa clustered together in the bootstrap test (1000 replicates) is shown next to the branches. The tree is drawn to scale with branch lengths in the same units as those of the evolutionary distances used to infer the phylogenetic tree.

**Figure 4:**
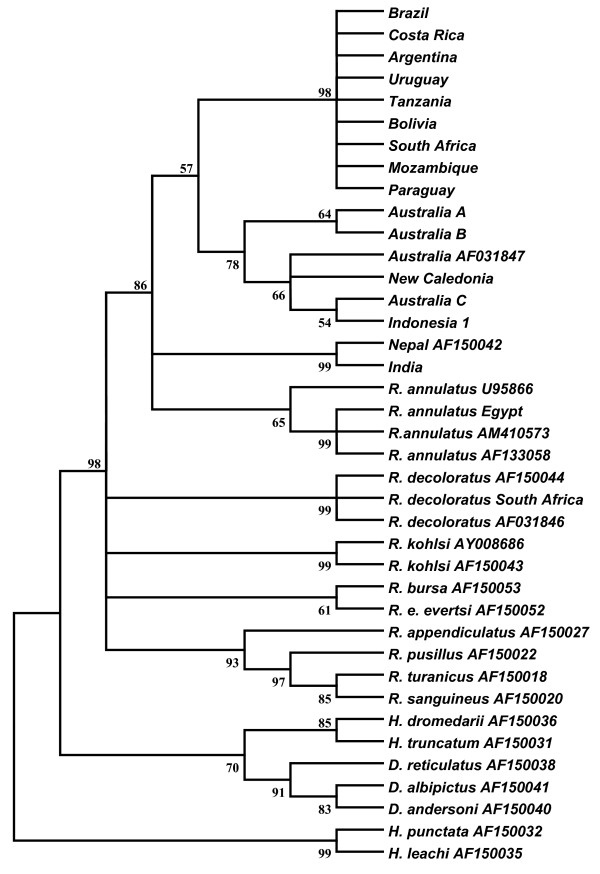
**Condensed Neighbor-Joining tree of the 12S rDNA sequences using the Tamura-Nei method**. The interior branches with less than 50% bootstrap support were collapsed. Numbers next to the branches represent the percentages of bootstrap values (1000 replicates).

The phylogenetic analysis of 16S sequences showed that sequences of *R. microplus *from America form a clade with African strains and the Taiwan (AY974232) sequence (98% bootstrap support) whereas the strains from Australia, New Caledonia and Indonesia grouped together in a different cluster (Fig. 3). Surprisingly, sequences of *R. annulatus *from USA and Spain formed a third cluster with *R. microplus *from India with a relatively high bootstrap support (76%) (Fig. 3). The phylogenetic analysis of 12S rDNA sequences was similar to the analysis of 16S rDNA (Fig. 4). This analysis provided strong support for a clade including *R. microplus *from America and Africa (98% bootstrap support). The strains from Australia, New Caledonia and Indonesia formed another cluster with a relatively high bootstrap support (78%). Finally, a strong support (99%) was found for a third clade containing *R. microplus *from India and Nepal (AF150043). In this analysis, the *R. annulatus *12S rDNA sequences grouped in a separate cluster with a low bootstrap support (65%).

**Figure 5:**
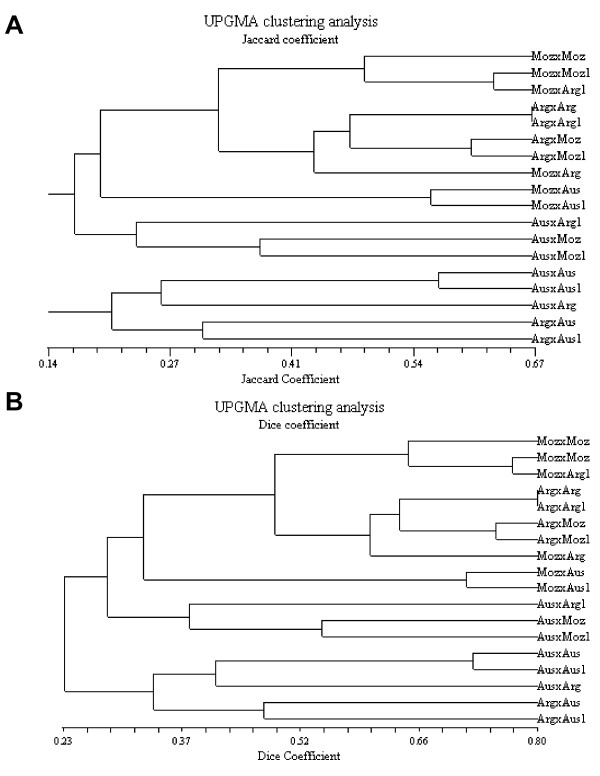
**UPGMA clustering analysis of microsatellite alleles obtained in the progenies of homologous and heterologous crosses of *R. microplus *tick strains from different geographic locations**. For homologous and heterologous crosses, female_strain 1 _× male_strain 2 _(Stran1 × Strain2) and female_strain 2 _× male_strain 1 _(Strain1 × Strain2 1) crosses were used. The similarity matrixes were obtained using (A) Jaccard's and (B) Dice's coefficients and the similarity values are shown in the X-axis. Both dendograms show the same topology.

The analysis of microsatellite loci was conducted to provide additional evidences of genetic polymorphism between tick strains using nuclear genome markers. In these experiments, egg masses from two separate homologous and heterologous crosses were used and allele number and size was determined for each locus (Table 5 and Additional file 1). The UPGMA clustering analysis of microsatellite alleles obtained in the progenies of *R. microplus *crosses showed that homologous and heterologous crosses involving AUS had lower similarity coefficients when compared to other homologous and heterologous crosses between MOZ and ARG strains (Fig. 5A and 5B). Some microsatellite loci did not provide a clear genotype for the MOZ × MOZ cross and in ticks from India and New Caledonia (Additional file 1), probably due to the presence of null alleles in these strains.

**Table 5:** Genotyping of microsatellite loci in geographic strains of *R. microplus*.

**Tick strains^a^**	**Microsatellite loci (repeated array)**											
	
	**BmA12** (CA)_3+7_(CG)_4_		**BmA06** (GT)_8+2_		**BmB12** (TA)_4_(TG)_9_		**BmC03** (CA)_10+9_		**BmC07** (GT)_17_		**BmD12** (CA)_10+5_	
	
	Na	SR (bp)	Na	SR (bp)	Na	SR (bp)	Na	SR (bp)	Na	SR (bp)	Na	SR (bp)
MOZ × MOZ	1	94	ND	ND	3	[180–216]	5	[157–171]	4	[132–192]	2	[93; 95]
ARG × ARG	1	94	2	[99; 101]	5	[180–216]	2	[173; 175]	1	144	1	93
AUS × AUS	4	[94–200]	4	[96–102]	4	[191–199]	4	[146–158]	3	[144–180]	4	[88–105]
MOZ × ARG	1	94	2	[99; 101]	4	[180–216]	6	[165–175]	5	[132–192]	5	[93–109]
AUS × ARG	4	[94–200]	5	[96–101]	6	[186–214]	5	[146–175]	5	[132–150]	8	[88–112]
AUS × MOZ	3	[94–198]	3	[98–102]	4	[180–199]	6	[157–171]	8	[144–192]	4	[88–109]
India	3	[198–218]	2	[108; 204]	1	180	ND	ND	ND	ND	ND	ND
New Caledonia	1	200	1	108	4	[176–199]	ND	ND	2	[146; 150]	1	107

^a^For Argentinean (ARG), Australian (AUS) and Mozambican (MOZ) strains, egg masses from two separate crosses were used for DNA extraction and genotyping following published procedures (22). For homologous and heterologous crosses, female_strain 1 _× male_strain 2 _and female_strain 2 _× male_strain 1 _crosses were used. For India and New Caledonia strains, pooled tick larvae and adult ticks were used for analysis, respectively. ND, not determined; Na, number of alleles; SR, allele size range.

## Discussion

This paper introduced a combined approach based on biological and molecular tools to provide additional support for the hypothesis of the existence of at least two different tick species currently considered as *R. microplus*. The most important evidences were provided by studies of crosses between tick strains which showed reproductive isolation of the Australian strain. Genetic analyses of mitochondrial and nuclear genome markers were not conclusive but provided support for the genetic divergence between the Australian and African/American strains.

Previous results have suggested that the Australian strains of *R. microplus *show biological differences when compared to other *R. microplus *strains from Africa and America [7]. In these studies, sterile progenies were obtained from crosses between strains from Australia and South Africa. In addition, the mean number of eggs produced by engorged females of American strains is higher than that obtained from Australian strains [27-30]. Furthermore, the amount of the insect growth regulator fluazuron required for complete growth inhibition in Australian tick strains is lower than that required for the Argentinean strains [31]. These results suggested that Australian *R. microplus *differ from strains in America and Africa and may have evolved into a separate species.

To test this hypothesis, we conducted experiments to characterize the reproductive performance and genetic diversity of *R. microplus *strains from Australia, Africa and America. The results clearly showed that tick strains from Africa (Mozambique) and America (Argentina) have no biological barrier for reproduction, being therefore the same species. All the reproductive parameters were significantly reduced when the Australian strain was involved in heterologous crosses suggesting the existence of a separate species. The genetic analysis of mitochondrial 12S and 16S rDNA as well as nuclear microsatellite loci sequences further suggested that strains from Africa and South America are conspecific. According to these analyses, ticks from Australia, Indonesia and New Caledonia would be also conspecific, different from the American-African strains. While clustered in a different clade, support for a separate species in India and Nepal was inconclusive. Interestingly, the results of the microsatellite analysis suggested the absence of transmission ratio distortion between paternal and maternal alleles even in the offspring of the AUS crosses with either ARG and/or MOZ strains, reducing the possibility of parental genetic effects to explain strain incompatibility.

It is well known that in the absence of males, Metastriata females increase their feeding period, have smaller repletion weights and oviposit none or only few fertile eggs when compared to females mated with conspecific males [32]. In the present study, females from all crosses showed feeding periods significantly shorter and significantly higher repletion weights than the virgin female controls. This result indicated that all crosses resulted in copula regardless of being fertile or not and evidenced that AUS females were attractive to ARG and MOZ males and *vice versa*. Attractiveness and cross-mating between *R. microplus *and *R. annulatus *has been reported, resulting in sterile hybrids [33], similarly to our results for heterologous crosses with AUS ticks.

One possible drawback of this study is the use of the Australian Yeerongpilly strain, which has been kept in the laboratory for over 60 years [34]. This strain has been also used in previous analyses of the reproductive success of heterologous crosses [7]. However, while low reproductive performance could be ascribed to continuous maintenance of the strain in the laboratory, differences were not observed in 12S and 16S rDNA sequences between the Yeerongpilly strain, the other two Australian strains analyzed in this study (A and B; Tables 4 and 5) and those reported by Murrell et al. [35]. Therefore, reproductive isolation seems to be a factual character associated to the strain and not a side effect of long-term laboratory maintenance.

Although mitochondrial 12S and 16S rDNA phylogenies were constructed with sequences from multiple Australian strains, the studies of the reproductive performance and microsatellite polymorphism were conducted with a single Australian (Yeerongpilly) strain, a fact that should be taken with caution when generalizing these results to Australian *R. microplus *in general. However, despite the limitations of the study, the results reported herein support the hypothesis that at least two species exist under the name *R. microplus*. These species could be redefined as *R. microplus *(Canestrini, 1887) (for American and African strains) and probably *R. australis *Fuller, 1899 (for Australian strains). After its description, *R. australis *was reported in Africa. However, these records were synonymized under *R. annulatus*. Interestingly, *R. annulatus *appeared within the monophyletic clade of *R. microplus *in the 16S mtDNA tree or as a paraphyletic branche in the 12S mtDNA tree suggesting that the denomination of *R. annulatus *may needs revision. Currently, *R. australis *is regarded as a synonym of *R. microplus*. Biological features reported here, including the lack of fertile F_1 _progeny in heterologous crosses and smaller, significantly different weight of engorged Australian (Yeerongpilly strain) females as well as molecular findings involving different Australian strains are clearly supportive of the hypothesis of a reproductive isolation between Australian and American/African ticks. We can not ascribe the Australian-Indonesian-Caledonian specimens to that species until the finding of its types and further morphological comparisons are conducted. It is also possible that other boophilid tick strains may inhabit small areas of the South Pacific. This issue warrants further studies by conducting detailed morphologic, genetic and reproductive studies with multiple tick strains collected at different geographic locations and cured from their endosymbionts which may affect tick reproductive performance.

It is important to note that the introduction of *R. microplus *into Africa took place with animals from India, after the epidemic of Rinderpest in the 19th century. Although it is hypothesized that *R. microplus *entered into South America along with Indian cattle, information is currently not available to support this hypothesis. However, the introduction of *R. microplus *in Australia took place with animals from Indonesia (most probably cattle from Java around 1870) [36] and then later into New Caledonia with Australian cattle (Barré, pers. comm.). This fact is in agreement with the reproductive and molecular findings of this study: ticks from Indonesia, Australia and New Caledonia may be a different, reproductively isolated species. As mentioned before, the strains from India and Nepal may constitute a different species, closer to African and American *R. microplus*, but adequate biological evidence is lacking and molecular evidence presented herein are not conclusive and/or may be the result of allopatric evolution. Detailed morphological, genetic and reproductive studies of tick strains from Africa, America, Australia, Indonesia, New Caledonia, India and Nepal would be necessary before confirming the redescription and possible resurrection of other boophilid tick species. Nevertheless, until further studies are conducted, caution should be taken in extrapolating results from studies on Australian ticks determined as *R. microplus*. These data have important implications in the field of animal health, because many studies involving ecology and control of cattle ticks performed on Australian tick strains considered as *R. microplus *may not be directly applicable to tick strains in America or Africa.

## Conclusion

The results reported herein provided biological and genetic evidences of allopatric speciation in *R. microplus *and suggested a reproductive isolation for Australian-Indonesian-Caledonian strains resulting in a different species. These results support the hypothesis that at least two different species have evolved under the name *R. microplus*. These species must be redefined after careful inspection of type specimens and further analysis of local tick fauna, including studies with several tick strains from each geographic location.

## Authors' contributions

MBL conducted the tick crosses. MBL and AE-P analyzed the results of tick crosses. VN and AJM obtained new tick sequences. AJM and AAG conducted phylogenetic analyses. VN, CT and JF obtained and analyzed tick microsatellites. MBL, AE-P, AAG, FJ and JF conceived and participated in study design. MBL, AE-P, AAG, AJM, FJ and JF helped to draft the manuscript. All authors read and approved the final manuscript.

## Supplementary Material

Additional file 1 **Microsatellite genotypes of individual crosses.** The data provided represent the microsatellite alleles identified in all tick crosses. Click here for file
